# A pilot study demonstrating the altered gut microbiota functionality in stable adults with Cystic Fibrosis

**DOI:** 10.1038/s41598-017-06880-y

**Published:** 2017-07-27

**Authors:** F. Fouhy, N. J. Ronan, O. O’Sullivan, Y. McCarthy, A. M. Walsh, D. M. Murphy, M. Daly, E. T. Flanagan, C. Fleming, M. McCarthy, C. Shortt, J. A. Eustace, F. Shanahan, M. C. Rea, R. P. Ross, C. Stanton, B. J. Plant

**Affiliations:** 1 Teagasc Food Research Centre, Moorepark, Fermoy, Co. Cork Ireland; 2 HRB Clinical Research Facility, University College Cork, Cork, Ireland; 3 Cork Cystic Fibrosis Centre, University College Cork, Cork University Hospital, Wilton Cork, Ireland; 4 APC Microbiome Institute, Cork, Ireland; 5 Department of Medicine, University College Cork, National University of Ireland, Cork, Ireland; 6 School of Microbiology, University College Cork, Cork, Ireland

## Abstract

Cystic Fibrosis (CF) and its treatment result in an altered gut microbiota composition compared to non-CF controls. However, the impact of this on gut microbiota functionality has not been extensively characterised. Our aim was to conduct a proof-of-principle study to investigate if measurable changes in gut microbiota functionality occur in adult CF patients compared to controls. Metagenomic DNA was extracted from faecal samples from six CF patients and six non-CF controls and shotgun metagenomic sequencing was performed on the MiSeq platform. Metabolomic analysis using gas chromatography-mass spectrometry was conducted on faecal water. The gut microbiota of the CF group was significantly different compared to the non-CF controls, with significantly increased *Firmicutes* and decreased *Bacteroidetes*. Functionality was altered, with higher pathway abundances and gene families involved in lipid (e.g. PWY 6284 unsaturated fatty acid biosynthesis (p = 0.016)) and xenobiotic metabolism (e.g. PWY-5430 meta-cleavage pathway of aromatic compounds (p = 0.004)) in CF patients compared to the controls. Significant differences in metabolites occurred between the two groups. This proof-of-principle study demonstrates that measurable changes in gut microbiota functionality occur in CF patients compared to controls. Larger studies are thus needed to interrogate this further.

## Introduction

Cystic Fibrosis (CF) is an autosomal recessive disorder affecting over 70,000 individuals globally^1, 2^. Caused by a mutation to the cystic fibrosis transmembrane conductance regulator (CFTR) gene, it results in an altered flow of chloride and bicarbonate ions across epithelial cells, the most significant result of which is the dehydration of the airway surface liquid layer in the lungs, resulting in the accumulation of thick mucus. As a result, bacteria can colonize this mucus layer more efficiently and develop into CF pathogens^3^. While the lungs are the most seriously affected organ in CF and unsurprisingly have received the greatest research focus to date, the CFTR protein is located throughout the body on the apical layer of the epithelial cells, thus resulting in multiple morbidities, including altered gastrointestinal functioning.

Multiple factors contribute to the altered gut microbiota in patients with CF including, the CFTR mutation resulting in increased mucus secretions in the small intestine^4^, altered diet and the use of pancreatic enzymes. Undoubtedly one of the most influential factors that results in alterations to the CF gut microbiota is the lifelong exposure to oral, inhaled and intravenous antibiotics^5^. The impact of antibiotics on the gut microbiota has been extensively studied in a non-CF cohort and shown to have significant effects, with recovery of the microbiota to the pre-treatment state often being slow or incomplete^6–9^. Studies using DNA based approaches, including next-generation sequencing, have shown the CF gut microbiota to be significantly different to the gut microbiota of non-CF controls^10, 11^. One study used DGGE fingerprinting to investigate the predominant faecal microbiota of paediatric patients with CF compared to their healthy non-CF siblings^12^. The results demonstrated that clostridia, *Bifidobacterium* spp., *Veillonella* spp. and *Bacteroides-Prevotella* spp. were consistently higher in siblings compared to those with CF. Additionally, a recent study identified the potential role of the CF mutation on the CF gut microbiota^13^. The study demonstrated that those who were homozygous-F508del had a more altered faecal microbiota compared to the other CF genotypes in the study cohort. Finally, the importance of understanding changes in the CF gut microbiota was reinforced in light of a recent study that demonstrated that changes to the gut microbiota may presage changes in lung microbiota^14^.

The majority of next-generation sequencing studies to date have relied on sequencing of the 16S rRNA bacterial gene. While these approaches have provided important insights into the CF lung^15, 16^ and gut microbiota^10^, the functionality of the altered CF microbiome has been poorly characterised. A recent study investigated the functionality of the CF lung microbiota using DNA and RNA sequencing^17^. However, there is a deficit of data regarding the CF gut microbiota, with no study examining functionality of the adult CF gut microbiota using shotgun metagenomic sequencing. A recent metagenomic study on the gut microbiota functionality of CF children (<3 years) demonstrated an altered functional capacity compared to non-CF children^18^. However, the authors suggested that this altered functionality was diminished with age. Thus, this pilot study wanted to take the first steps to investigate if in fact there would be measurable changes to the gut microbiota functionality in an adult CF population. Moreover, we wanted to investigate the metabolite profile of adult CF patients compared to controls. Our results showed significant changes in the functional diversity of the CF gut compared to the controls. Additionally, metabolites and gut microbiota were significantly altered in the CF group compared to the non-CF controls.

## Results

### Functional analysis

We studied the microbiota composition and functionality of the CF patients compared to the controls, using the HUMAnN2 analysis pipeline.

**Figure 1:**
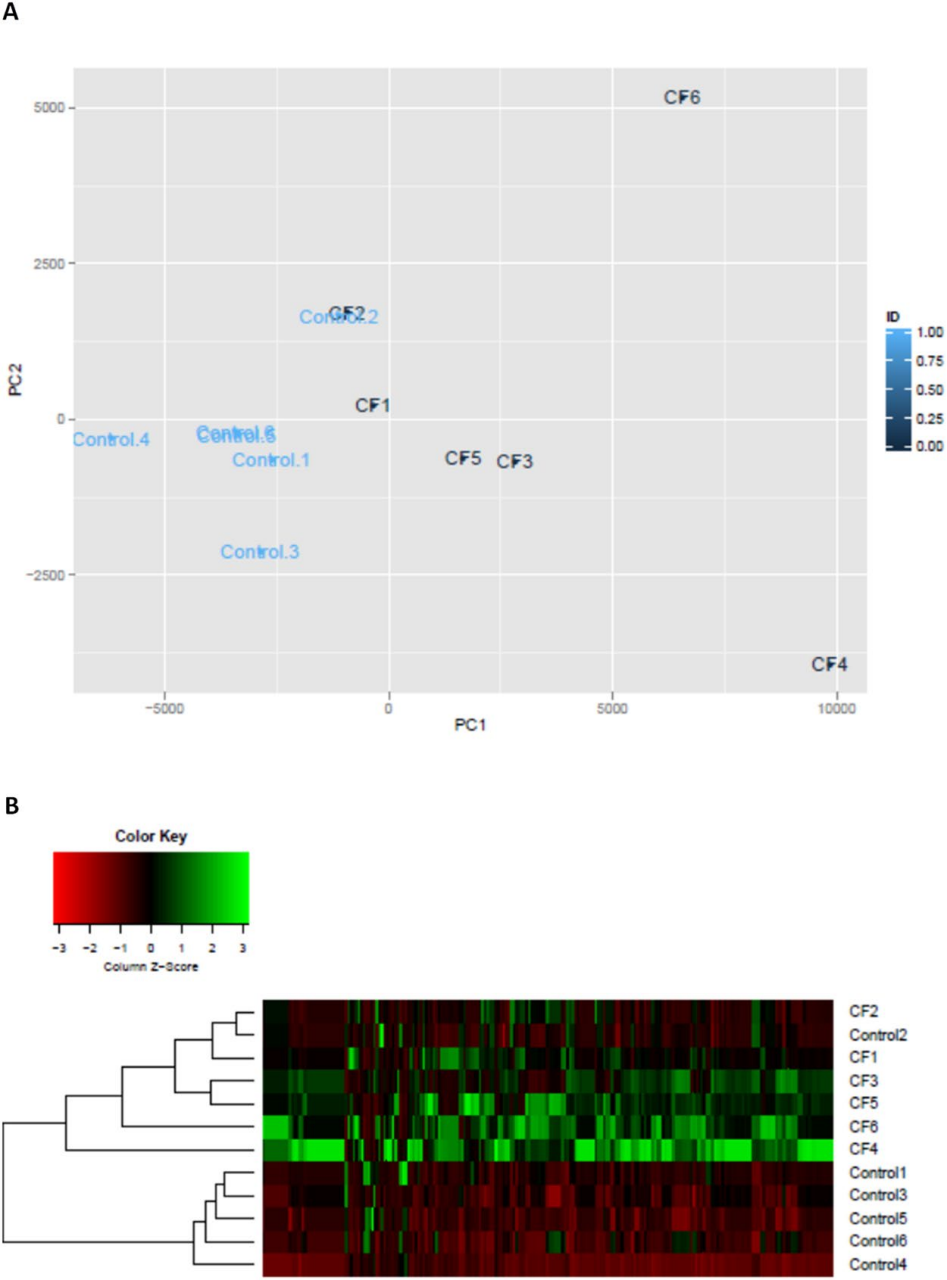
PCA plot (**A**) and a heat map (**B**) of pathway abundances based on pathways that were significantly altered between the two groups.

Of the 2066 pathways, 10.7% were found at significantly different abundances in the CF group compared to the controls. A PCA plot was also generated on the relative abundances of pathways in the CF and control patients (Fig. 1A). The plot showed clustering of the CF samples distinctly from the controls. A heat map demonstrated similar clustering of controls from CF patients based on pathway abundances (Fig. 1B), though in this instance, CF2 was more similar to the controls than the CF patients. Two of the six CF patients (CF4 and CF5) were pancreatic sufficient. However, they did not cluster more closely together or appear more similar to each other compared to the four pancreatic insufficient patients. Diversity analysis on the pathway abundances was completed on both groups, with significantly lower pathway abundance diversity in the controls compared to the CF group (Shannon Index p = 0.01).

### Lipid metabolism pathways

Pathways involved in unsaturated fatty acid biosynthesis (PWY 6284) (p = 0.016), fatty acid biosynthesis (PWY 6285) (p = 0.025) and palmitoleate biosynthesis (PWY 6282) (p = 0.025) were significantly increased in the CF patients. Furthermore fatty acid elongation FASYN-ELONG-PWY (p = 0.025), fatty acid oxidation (PWY 2501) (p = 0.01), super pathway of fatty acid biosynthesis PWYO-881 (p = 0.025) and fatty acid and β-oxidation V PWY66–391 (p = 0.05) were all significantly increased in CF patients compared to controls. Using the pathway abundance data from HUMAnN2 we identified in which bacteria the pathways were found in and their relative abundance. From this information, we could ascertain pathways that were present in a higher abundance in CF patients compared to controls, due to the fact that a limited number of bacteria were increased in CF patients compared to controls. It was apparent that increased *Ruminococcus*, *Clostridia*, *Enterococcus* and *Eggerthella* resulted in the increased pathway abundances compared to the controls, thus signifying even small changes in microbiota can have a measurable impact on microbiota functionality.

### Protein metabolism

The gut microbiota of the CF group exhibited several significant alterations to pathways involved in protein metabolism compared to the controls. The pathway abundances of arginine degradation ARGASEDEG-PWY (p = 0.01), homomethionine biosynthesis PWY 1186 (p = 0.025), tryptophan degradation pathway V PWY 3162 (p = 0.006) and L cysteine biosynthesis PWY 7289 (p = 0.05) were all significantly increased in those with CF compared to the controls.

### Carbohydrate metabolism and pentose phosphate pathway

With respect to carbohydrate metabolism, the lactose utilization pathway (LACTOSEUTIL-PWY) was increased in the gut microbiota of those with CF compared to controls (p = 0.025). This could be seen with an increased abundance of this pathway in *Ruminococcus gnavus* (p = 0.014), *R. torques* (p = 0.007), *C. clostridioform* (p = 0.005) and *E. faecalis* (p = 0.007) in CF compared to controls. Additionally, starch degradation pathway (IV) PWY-6735 (p = 0.006) and pathway I PWY-842 (p = 0.010) abundances were significantly increased in the gut microbiota of the CF group compared to the controls. It was noted that the levels of these pathways in *Bacteroides, Lachnospiracheae, Clostridium and Enterococcus* were significantly increased in the CF patients compared to the controls, which could contribute to the altered starch catabolic pathways.

There were significant changes to the pentose phosphate pathways. Pentose phosphate pathway (oxidative branch II) OXIDATIVEPENT-PWY-1 was significantly increased in abundance in the CF patients compared to the controls (p = 0.016). Additionally, the non-oxidative branch of the pentose phosphate pathway NONOXIPENT-PWY was increased (p = 0.037) compared to the controls.

### Xenobiotic degradation

Given the higher exposure of individuals with CF to antibiotics and therapeutic medicines compared to non-CF controls, we were interested in the impact this may have on the gut microbiota and the potential increases in bacteria capable of degrading xenobiotic compounds. The results showed that there were significantly increased abundances of pathways involved in xenobiotic metabolism in the CF gut microbiota compared to the controls, including PWY-5178 toluene degradation IV (p = 0.004), PWY-5430 meta cleavage pathway of aromatic compounds (p = 0.004), 4TOLCARBDEG-PWY 4 tolenecarboxylate degradation (p = 0.025) and PWY-5179 toluene degradation V (aerobic) (p = 0.028).

### Folate metabolism and antibiotic biosynthesis

Some interesting non-significant increases in pathway abundances were also noted namely in folate metabolism and antibiotic biosynthesis. There was a non-significant trend of increase in folate transformations in the CF gut microbiota compared to the controls. We also noted there were higher abundances of antibiotic biosynthesis pathways in those with CF compared with controls. These included gentamicin biosynthesis PWY-7025 (5066 vs. 4594 in CF vs. control gut microbiota), neomycin PWY 7016 (13.7 vs. 9.17 CF vs. control), paromycin biosynthesis PWY 7018 (1891 vs. 1575 CF vs. control), fosfomycin biosynthesis PWY 5757 (3.45 vs. 1.92 in CF vs. control gut), kanamycin biosynthesis PWY 7000 (3.34 vs. 2.75 CF vs. control) and streptomycin biosynthesis PWY 5940 (79.0 vs. 68.5 CF vs. control gut).

### Gene abundance analysis based on gene ontology (GO)

#### Metabolic functions

**Figure 2:**
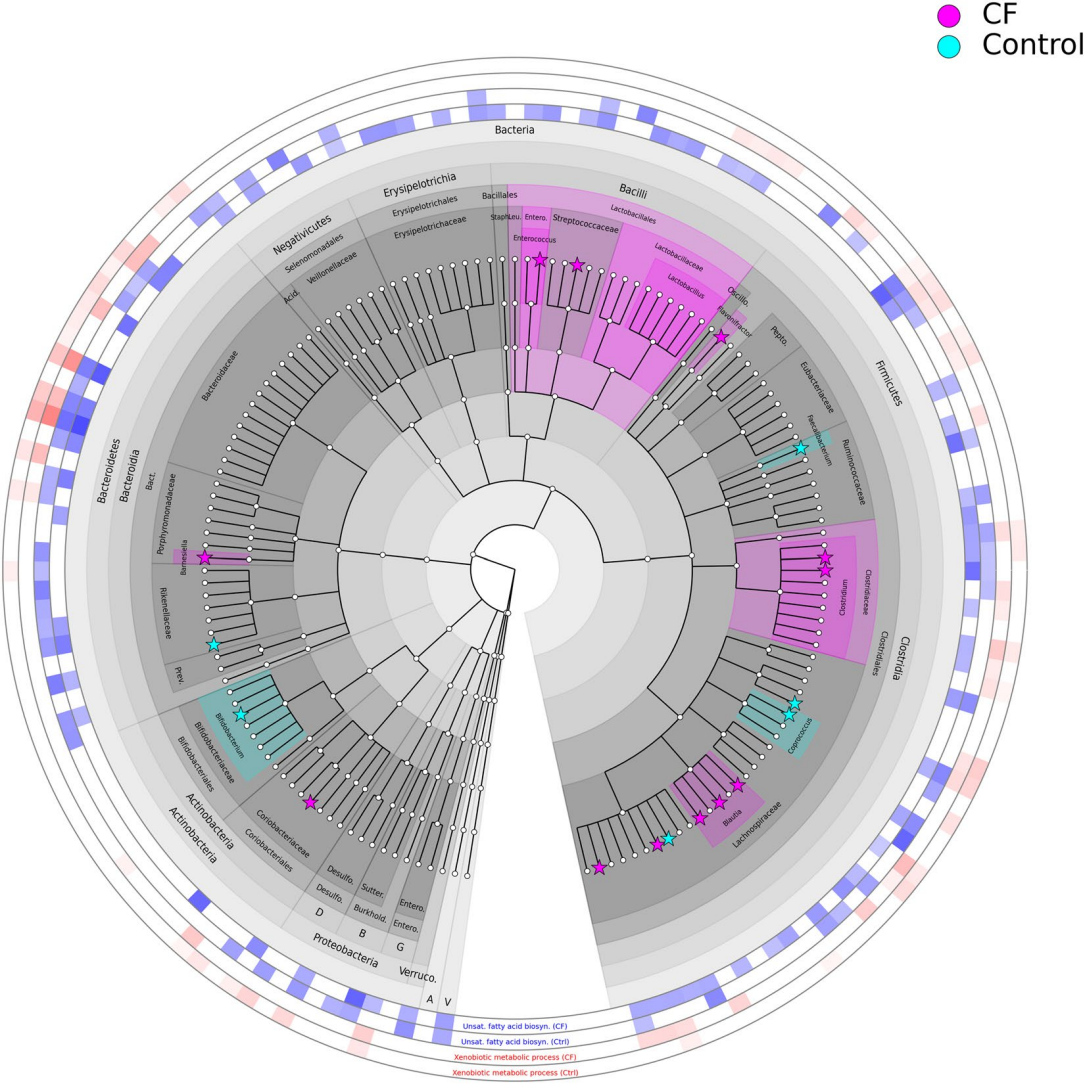
Cladogram presenting the microorganisms detected in stool samples from CF patients and controls using MetaPhlAn2, and the relative contribution of each microbial species to unsaturated fatty acid biosynthesis and xenobiotic metabolic processes, as determined by HUMAnN2. Pink clades indicate that taxa were significantly higher in CF patients whereas cyan clades indicate that taxa were significantly higher in controls. Stars are used to highlight species that were significantly different between the two groups. The outer rings indicate the abundance of unsaturated fatty acid biosynthesis pathways (blue) and xenobiotic metabolic process pathways (red) that was assigned to each species by HUMAnN2.

With respect to metabolic functions, the relative abundance of 109 functions out of 654 (16.7%) were significantly altered between the groups. Interestingly, the relative abundance of only two metabolic functions were increased in the control group compared to the CF group - namely GO: 0004717 transmembrane receptor protein tyrosine kinase and GO: 00018024 histone-lysine N-methyltransferase activity. Some notable increased gene family abundances occurred in the gut microbiota of the CF group compared to controls, including genes involved in antibiotic resistance. These included O acetlytransferase activity (involved in acetylation of aminoglycosides conferring resistance to the bacterial host) (GO: 0016413; p = 0.006), drug transmembrane transporter activity (GO: 0015238; p = 0.01), porin activity (GO: 0015288; p = 0.025) and penicillin binding (GO: 0008658; p = 0.037). Using bacterial species information, we examined the bacteria responsible for the significantly increased abundance of these gene families in the CF gut compared to the controls. It was found that significantly higher levels of these pathways occurred in *Clostridium*, *Blautia*, *Lachnospiraceae*, *Bacteroides* and *Enterococcus*, while extremely low levels or absence of these gene families in these bacteria occurred in the control group, owing to decreased levels or absence of these bacteria and their associated pathways in the control group (Fig. 2).

### Cellular components and biological processes

With respect to gene families involved in cellular components, 13% were significantly increased in the CF group compared to the controls. These included significant increases in nucleosome (GO: 0000786; p = 0.037), plasma membrane (GO: 0005886; p = 0.016) and bacterial type flagellum (GO: 0009288; p = 0.037).

To investigate changes in biological processes, we looked at amino acid, lipid and carbohydrate metabolism, as well as xenobiotic degradation (which was of particular interest within a CF cohort due to their chronic exposure to antibiotics and pharmaceuticals from an early age). With respect to protein metabolism, 278 processes were identified, of which 16 were significantly increased and just one was decreased in the gut microbiota of the CF group compared to the controls (significant results shown in Supplementary Table S3). The results confirm findings of the increased pathway abundances including increased tryptophan and arginine metabolism (GO: 0000162; p = 0.037, GO: 0006568; p = 0.037, GO: 0010121; p = 0.037).

Of the 268 gene families involved in lipid metabolism, 22 were significantly different between the CF and control groups, five of which were increased in the controls compared to the CF group (Supplementary Table S3). The results indicated that the gut microbiota of individuals with CF has enhanced abundances of genes contributing to lipid metabolism including fatty acid biosynthesis (GO: 0006633; p = 0.016), fatty acid elongation (GO: 0030497; p = 0.025) and methyl-branched fatty acid metabolic processes (GO: 0097089; p = 0.022).

Seventy-two of the 276 gene families involved in carbohydrate metabolism were significantly different between the two groups, the majority of which (75.4%) were significantly increased in the gut microbiota of the CF group compared to the controls (Supplementary Table S3). These included polysaccharide biosynthetic processes (GO: 0000271; p = 0.025), starch catabolic processes (GO: 0005983; p = 0.025) and pectin metabolic processes (GO: 0045488; p = 0.037). Due to the higher exposure to and chronic use of antibiotics and other pharmaceutical therapeutics in the treatment of CF, we were interested in investigating changes in the abundance of genes involved in xenobiotic degradation. We found four significant xenobiotic metabolic processes in the CF group compared to the controls (Supplementary Table S3) including enhanced xenobiotic metabolic (GO: 0006805; p = 0.01) and catabolic processes (GO: 0042178; p = 0.01).

**Figure 3:**
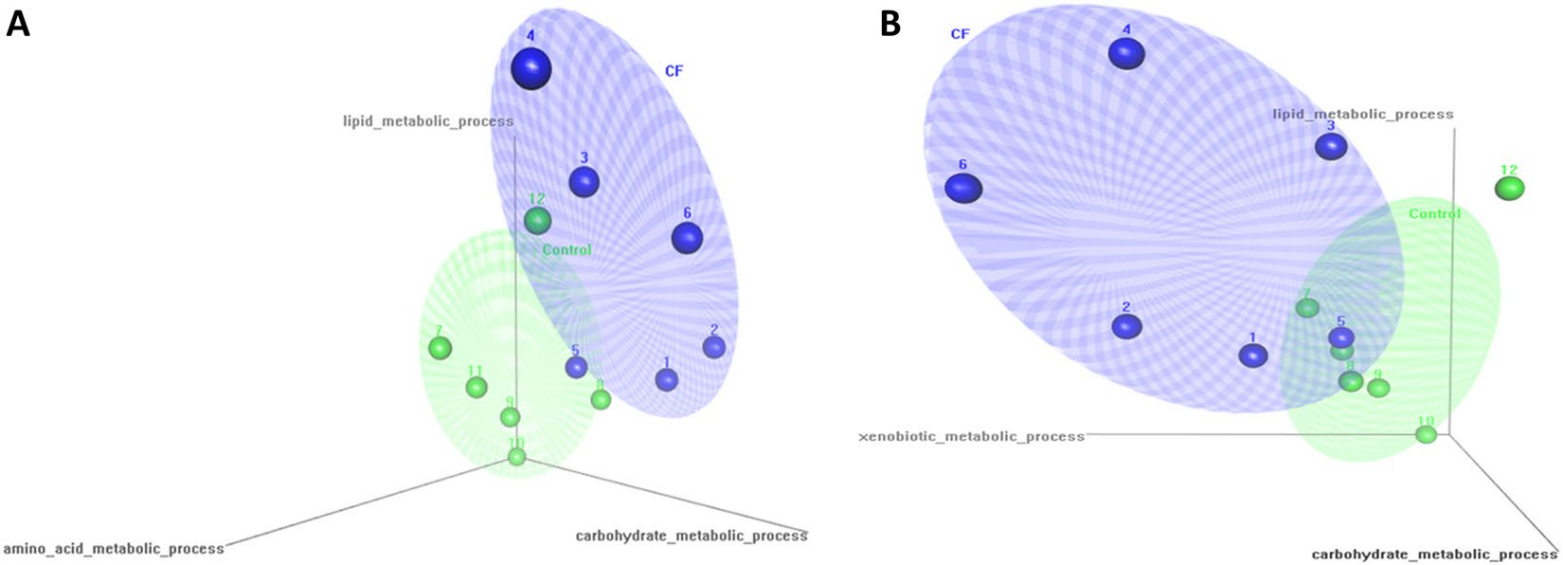
3D Scatter plot of the CF and control samples using the gene abundance data and the GO terms lipid metabolism, carbohydrate metabolism and amino acid metabolism as the principle coordinates. x-axis = GO:0005975|BP|03| carbohydrate metabolic process, y-axis = GO:0006629|BP|03| lipid metabolic process, z-axis = GO:0006520|BP|03|cellular amino acid metabolic process (Panel A) or xenobiotic metabolism z-axis = GO:0006805|BP|03|xenobiotic metabolic process (Panel B).

To examine the combined effect of these changes to gene families, we used the GO terms to create a 3D scatter plot of our two groups. Initially (Fig. 3A) we used lipid (GO: 0006629), carbohydrate (GO: 0005975) and protein metabolism (GO: 0006250) as the three principle axes. The results show that the CF and control samples cluster separately, with only a slight overlap of the two clusters. The CF samples were higher in lipid and carbohydrate metabolism gene families, while the controls located closer to the protein metabolism axis. To investigate the effect of xenobiotic metabolic processes, we replaced the amino acid metabolism axis with xenobiotic metabolic processes (GO: 0006805) (Fig. 3B). Again, two clusters formed with the CF and the control samples clustering separately. The CF samples were higher in lipid and xenobiotic metabolism gene family abundances.

### Phylogenetic investigations

**Figure 4:**
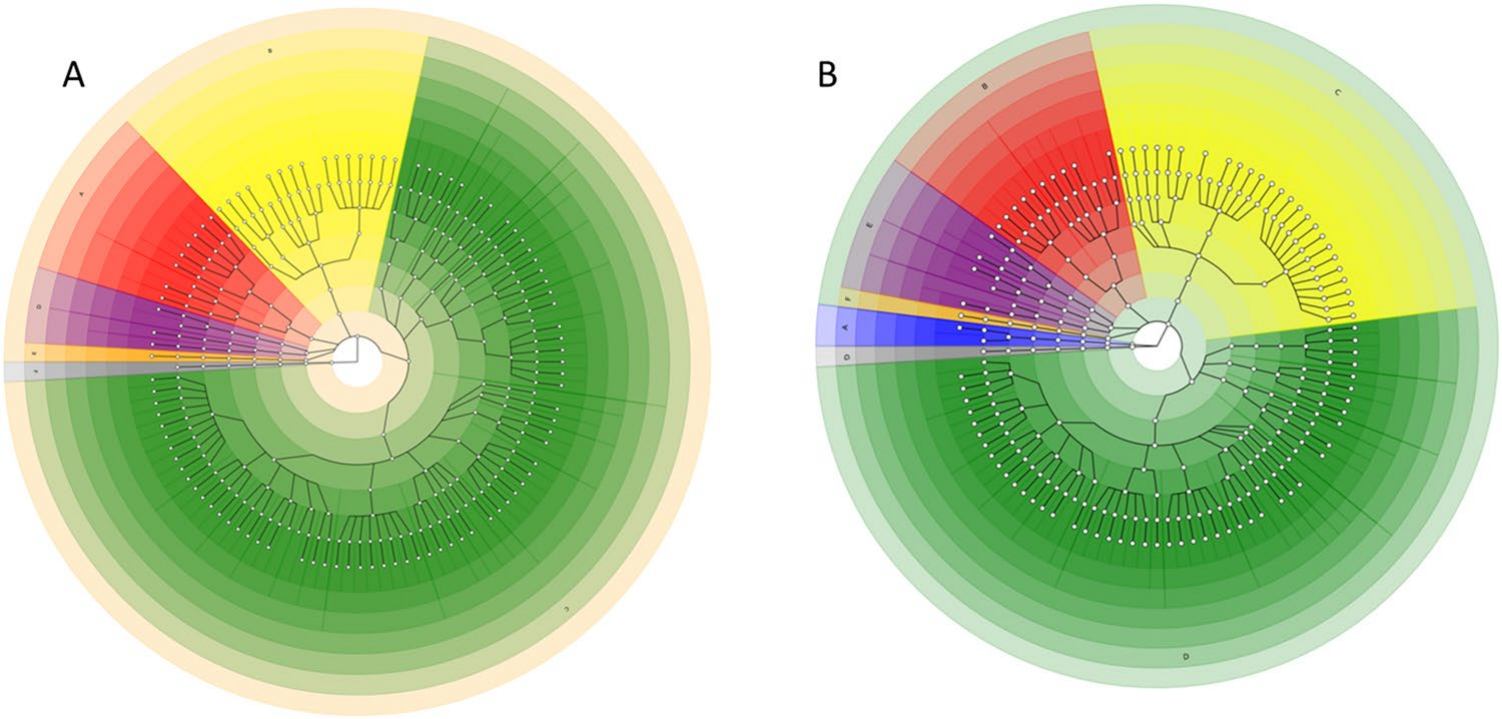
Graphlan generated phylogenetic tree of 16S rRNA based phylogenetic composition of the CF (**A**) and control (**B**) groups. CF legend: A: *Actinobacteria* (red), B: *Bacteroidetes* (yellow), C: *Firmicutes* (green), D: *Proteobacteria* (purple), E: *Verrucomicrobia* (orange), F: *Viruses* (grey). Control legend: A: *Archaea* (blue), B: *Actinobacteria* (red), C: *Bacteroidetes* (yellow), D: *Firmicutes* (green), E: *Proteobacteria* (purple), F: *Verrucomicrobia* (orange), G: *Viruses* (grey).

To investigate the phylogenetic composition of our two groups, we extracted taxonomic data using MetaPhlan from the metagenomic data. Using the Graphlan package we analysed the phylogenetic composition of the two groups^19, 20^. Figure 4 shows the microbial populations present in the CF (A) and control (B) groups. The results indicate significant changes to the microbiota present in the CF group compared to the controls. The CF group had increased *Firmicutes*, decreased *Actinobacteria*, *Bacteroidetes* and *Proteobacteria* compared to the controls. *Archaea* were only detected in the control group. Levels of viruses and *Verrucomicrobia* were comparable between the two groups.

When statistical analysis was performed on the relative abundance of bacteria present, there were notable statistically significant differences between the gut microbiota in both groups (Table 1). At genus level, *Bifidobacterium* (3.21 (CF) vs. 6.81% (control) relative abundance; p = 0.05) and at species level, *B. longum* was significantly decreased in the CF group (2.12 vs. 4.32% relative abundance; p = 0.046) compared to the controls. With respect to the changes within the phyla *Bacteroidetes*, *Barnsiella intestinihominis* was absent in the CF group compared to the controls (p = 0.007). The greatest number of differences between the groups was seen within the *Firmicutes* phylum. Of particular note was the significantly higher levels of *Enterococcus faecalis* (8.83 vs. 0% relative abundance p = 0.007) compared to the controls (in whom this bacterium was not detected). Additionally, significant increases in *Clostridium* were seen in the CF group (p = 0.004) and *C. hathewayi* was only detected in the CF gut microbiota and not in the controls. There was a significant decrease in *Faecalibacterium prausnitzii* in the CF group compared to the controls (p = 0.003), while *Ruminococcus gnavus* and *R. torques* were significantly increased in the CF group (p = 0.022 and 0.013 respectively). Additionally, *Blautia* and *Lachnospiracheae* were only detected in the CF samples and not in the controls. Such changes in microbiota reflect the altered pathway abundances between the groups, as altering the microbiota prevalence results in altered abundance of their associated pathways.

**Table 1:** Relative abundances of bacteria found at significantly different levels in the CF and control groups.

Phylogeny	CF average relative abundance	Control average relative abundance	P value (corrected)
**Actinobacteria**			
*Bifidobacterium*	3.20967333	6.81271667	0.05
*Bifidobacterium longum*	2.12066833	4.23268333	0.046
*Bifidobacterium longum*_unclassified	2.12066833	4.23268333	0.046
*Eggerthella lenta*	0.19998833	0.00330333	0.013
*Eggerthella lenta*|t__GCF_000024265	0.19998833	0.00330333	0.013
**Bacteroidetes**			
*Barnesiella*	0	1.514735	0.007
*Barnesiella intestinihominis*	0	1.514735	0.007
*Barnesiella_intestinihominis*|t__GCF_000296465	0	1.514735	0.007
*Alistipes putredinis*	0.05026667	4.24946833	0.05
*Alistipes putredinis*|t__GCF_000154465	0.05026667	4.24946833	0.05
**Firmicutes**			
*Bacilli*	12.81672	0.78161167	0.016
*Lactobacillales*	12.456185	0.78161167	0.016
*Enterococcaceae*	9.54589833	0	0.007
*Enterococcus*	9.54589833	0	0.007
*Enterococcus faecalis*	8.832115	0	0.007
*Enterococcus faecalis*_unclassified	8.832115	0	0.007
*Lactobacillaceae*	1.59546167	0	0.022
*Lactobacillus*	0.483995	0	0.022
*Streptococcus thermophilus*	1.27870833	0.10655	0.049
*Streptococcus thermophilus*_unclassified	1.27870833	0.10655	0.049
*Clostridiaceae*	11.9238667	0.50407667	0.004
*Clostridium*	11.9238667	0.48078667	0.004
*Clostridium clostridioforme*	6.65942167	0.11350667	0.022
*Clostridium clostridioforme*_unclassified	6.65942167	0.11350667	0.022
*Clostridium hathewayi*	0.29948667	0	0.007
*Clostridium hathewayi_*unclassified	0.29948667	0	0.007
*Clostridiales* _noname	0.15274667	0.00104167	0.013
*Flavonifractor*	0.14014	0.00104167	0.05
*Flavonifractor plautii*	0.14014	0.00104167	0.05
Flavonifractor plautii GCF_000239295	0.14014	0.00104167	0.05
*Anaerostipes*_unclassified	0.28471833	0	0.007
*Blautia*	10.2605633	0.89512667	0.016
*Blautia producta*	0.77242	0	0.022
*Blautia producta*|t__GCF_000373885	0.77242	0	0.022
*Ruminococcus gnavus*	8.05991833	0.38045667	0.022
*Ruminococcus gnavus*_unclassified	8.05991833	0.38045667	0.022
*Ruminococcus torques*	1.07679	0.054245	0.013
*Ruminococcus torques_*unclassified	1.07679	0.054245	0.013
*Coprococcus*	0.17310667	4.51670167	0.033
*Coprococcus comes*	0.13519667	2.253635	0.021
*Coprococcus comes*|t__GCF_000155875	0.13519667	2.253635	0.021
*Dorea formicigenerans*	0.169365	0.73247667	0.049
*Dorea formicigenerans*_unclassified	0.169365	0.73247667	0.049
*Lachnospiraceae bacterium*_1_1_57FAA	0.40623667	0	0.007
*Lachnospiraceae bacterium*_1_1_57FAA|t__GCF_000218445	0.40623667	0	0.007
*Lachnospiraceae bacterium*_5_1_63FAA	0.01507333	0.414975	0.05
*Lachnospiraceae bacterium*_5_1_63FAA|t__GCF_000185525	0.01507333	0.414975	0.05
*Lachnospiraceae bacterium*_8_1_57FAA	0.15276667	0	0.022
*Lachnospiraceae bacterium*_8_1_57FAA|t__GCF_000185545	0.15276667	0	0.022
*Faecalibacterium*	0.06931833	3.33589167	0.003
*Faecalibacterium prausnitzii*	0.06931833	3.33589167	0.003
*Faecalibacterium prausnitzii*_unclassified	0.06931833	3.33589167	0.003

### qPCR to investigate the microbial load in the CF and control gut microbiota

To verify that the phylogenetic changes are not indicative of alterations in the total microbial load in the CF gut compared to the controls, qPCR analysis was conducted on total bacterial DNA from all study participants. The qPCR analysis found no significant differences in 16S rRNA gene copy number/g faeces between the two groups (1.59 × 10^8^ (CF) vs. 3.07 × 10^8^ (control); p = 0.150), thus alterations in the microbiota do not result from differences in total bacteria load between the two groups.

### GC-MS results

**Figure 5:**
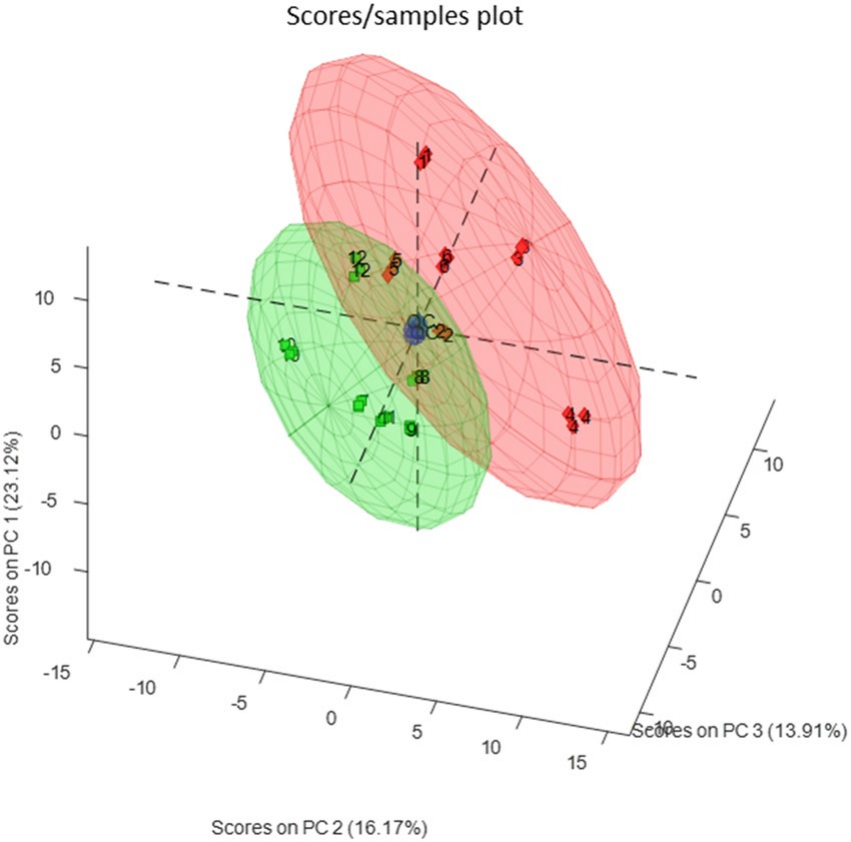
Score plot from PCA model calculated on the relative concentrations of the variables in the reduced dataset. Green: control samples. Red: CF samples. The blue samples in the centre are the QC samples. Data has been auto scaled.

To investigate the impact of an altered gut microbiota on metabolites, faecal water from the two groups was analysed using GC-MS. In the PCS model, the first three principle components all contribute to a partial separation of the groups, with the CF (red) and the control (green) samples forming distinct clusters (Fig. 5). Some overlap between the groups is seen, indicating some shared metabolites between CF patients and controls. Using the unsupervised PCA model, there was a clear tendency towards a grouping of two classes. The supervised method PLS-DA was used in an effort to find common characteristics for the two sample groups. The resulting predictions on the test are shown in Supplementary Fig. S1, where the lower plot shows the predictions of the sample. Samples with values higher than 0.5 are predicted to be from CF samples and those with values lower than 0.5 are predicted to be from the controls. As can be seen in Supplementary Fig. S1, all samples were correctly classified. This indicates that it is possible to find a set of common characteristics between the CF and control samples respectively. Variables with values above zero in the regression vector tend to be high in CF samples, while those lower than zero tend to be low compared to the control samples.

Statistical analysis was carried out on the non-parametric relative concentrations for each metabolite from the CF and control samples. Of the 99 metabolites, 16 were significantly different between the groups, with 15 of these being significantly higher in the CF samples compared to the controls. Significant increases were seen in hexadecanoic acid (p = 0.037), myristic acid (p = 0.037) and 9-octadecenoic acid (p = 0.025) in the CF samples compared to the controls (Supplementary Table 4). To elucidate similarities in clustering of metabolomic and metagenomic data, Procrustes and co-inertia analysis were performed (Supplementary Fig. S2). A strong positive correlation (r^2^ = 0.81) was observed using both co-inertia and Procrustes analysis between the metabolites and the pathway abundances, indicating similar clustering of both metagenomic and metabolomic datasets.

## Discussion

Cystic Fibrosis was first studied as a gastrointestinal disease and was only recognised as a separate disease to coeliac disease in 1938^21^. Historically, it was a disease characterized by malabsorption of fats and proteins, resulting in steattorhea and pulmonary infection^3^. Since 2011, the gut microbiota in CF has received renewed attention^10–13^, with studies showing alterations in the CF gut microbiota compared to controls. However, research remains limited, particularly on what impact such changes have on functionality and to date only one shotgun metagenomic study has been completed on the gut microbiota in children with CF, with no adult CF shotgun metagenomic study being reported. Our aim was to conduct a proof-of-principle study to investigate if there would be measurable changes to the gut microbiota functionality in an adult CF population.

One of the key findings from our pilot study was the enhanced potential for fat metabolism in the CF gut microbiota compared to the control group. Pathway and gene family abundances associated with fatty acid biosynthesis, fatty acid elongation, and fatty acid metabolism were all significantly increased in the CF gut microbiota compared to the controls. Additionally, the metabolomic results showed higher levels of several fats in the CF metabolite profile compared to the controls including octadecanoic acid, hexadecanoic acid and myristic acid. It is possible that the enhanced potential of the CF gut microbiota to synthesise and metabolise lipids could contribute to the increased fats seen in the metabolomics data from the CF group. However, it is important to note, reduced fat absorption occurs in patients with CF and thus a high fat diet is required to ensure adequate weight maintenance. Thus, it is likely that the increased fat metabolites occur as a result of a combination of factors including an altered diet, reduced absorption and an altered gut microbiota.

Another finding of our study was the potentially enhanced ability of the CF gut microbiota to metabolise xenobiotic compounds compared to the controls. Additionally, pathways and gene families involved in resistance to antibiotics were found in higher abundances in the CF group compared to the controls. These include porin activity, penicillin binding, and response to antibiotic changes. Given the high exposure of CF patients to antibiotics throughout their lifespan, there is continuous opportunity for the gut microbiota to acquire and develop resistance genes and pathways. The CF cohort in this study were exposed to oral, IV, inhaled and long term maintenance antibiotics in the 12 months prior to sample collection. This cohort is highly reflective of adult CF patients and their typical antibiotic exposure over a 12 month period. Thus they have extensive and prolonged exposure to different antibiotics, the majority of which are broad spectrum. By comparing the results from the CF group to those from the non-CF control (who had no antibiotic exposure for at least 28 days prior to sampling), we could investigate the impact of a lifetime of antibiotic exposure on gut microbiota composition and functionality. Several studies have demonstrated that the human gut microbiota acts as a reservoir of resistance and it is most probable that the greater the exposure to antibiotics, the greater the pressure to select for resistant microorganisms^22–24^. This was also highlighted in a recent study that demonstrated higher amoxicillin-clavulanic acid resistant *Enterobacteriaceae* in the faeces of individuals with CF compared to their non-CF siblings^25^. They also suggested that more frequent exposure to this antibiotic in those with CF leads to the development of resistant bacteria in the gut microbiota. The higher abundances of these xenobiotic metabolism and antibiotic resistant genes were found in a limited number of microorganisms, which caused the differences between the two groups. These included *Lachnospiracheae*, *E. faecalis*, *Clostridium* and *Bacteroides*. It is interesting to note that *E. faecalis* and *C. hathewayi* were only identified in the CF gut microbiota and not in the controls. Enrichment of *E. faecalis* levels in CF children has previously been shown^18^. The antibiotic resistance of *E. faecalis* is widely recognised and has received considerable attention as one of the major threats to human health as a consequence^26–28^, while *C. hatewayi* has also been linked to human bacteremia and infections^29, 30^. Detecting increased abundances of resistance pathways/genes in these bacteria in the CF gut is unsurprising, but of concern, and likely reflects the chronic exposure to antibiotics and their effects on gut microbiota. These results highlight the need for further studies ideally investigating such changes in functionality across periods of pulmonary stability, exacerbation and post-exacerbation. We hope to conduct such studies, powered based on our current data.

The results also showed significant changes in pathways and gene abundances involved in amino acid metabolism between the groups. These included increased arginine and tryptophan metabolic pathways. This suggests an enhanced ability of the gut microbiota of individuals with CF to metabolise proteins. It has been well documented that there is increased catabolism of proteins in CF individuals, leading to excessive energy losses^31^. These results suggest that the altered gut microbiota could potentially add to increased protein catabolism, though proof of this is required with transcriptomic and proteomic data. However, our findings are consistent with those of a recent study that used faecal proteomics to investigate alterations in gut microbiota and inflammation in the CF gut^32^. The study of 15 children with CF and their unaffected siblings found significant changes in faecal proteins in the CF cohort, with a dominance of proteins involved in inflammation and mucus formation in the CF group. These changes were associated with changes in faecal microbiota, with increased *Enterobacteriaceae* and *Clostridium* species and a reduction in *F. prausnitzii o*ccurring in the CF group. As was seen in our study, increased *Clostridium clostridioforme* and *Ruminococcus gnavus* was found in the CF faecal samples. Significant reductions of *F. prausnitzii* observed in that study and ours are consistent with previous findings^33^. Similarities between our adult findings and those of previous studies on CF children^18^, suggest that the altered gut microbiota composition and functionality that occurs in children with CF is maintained through to adulthood in CF patients. This demonstrates the need for further investigations in larger adult CF studies going forward.

Significant differences also occurred in the metabolite profiles between the 2 groups and we were able to accurately predict if a sample was from the CF or control group, based on the metabolite profile alone. However, it was not possible in this pilot study to determine which particular metabolites act as a marker of CF, rather a combination of metabolites appear to occur as a CF signature. There would be merit in further studying this with a larger cohort, having established here that different metabolite profiles occur between CF and control cohorts. Additionally, it would be interesting to study how the metabolite profile shifts from stability through exacerbation and into the post-exacerbation period. This could help identify if changes in the gut microbiota and faecal metabolites could predict exacerbation onset or determine the effects of exacerbation and its treatment on metabolite profile.

Ours is the first study to investigate the impact of changes in gut microbiota on functionality in adult CF patients using shotgun metagenomics and metabolomics. Previous studies on sputum showed significant variability in the lung microbiota between patients with CF, though the overall functionality remained similar^17^. Additionally, previous studies have shown the human gut microbiota to have considerable redundancy, thus functionality remains similar despite large inter-individual variations in microbiota^34, 35^. However, our CF gut microbiota study showed that both the microbiota composition and functionality was significantly different between the CF and control groups. Manor *et al*. previously demonstrated that the gastrointestinal microbiome of young children with CF has an altered functional capacity compared to non CF children, but suggested that this was diminished with age^18^. However, our results demonstrate that there are still measurable changes in gut microbiota functionality in an adult CF cohort compared to non-CF controls. Due to the multiple factors that can alter gut microbiota in individuals with CF (diet, genetics, antibiotic exposure, pancreatic enzyme use, hospitalization), it is not surprising that functionality is also impacted upon, as demonstrated in this study.

Despite the relatively limited sample size in this study, this study demonstrates using in-depth shotgun sequencing and metabolomics data, that clear changes occur at both phylogenetic and functional levels between the gut microbiota within the two groups. Our study was only conducted on stable CF patients. However, studies conducted on stable and exacerbating CF patients would enhance our understanding of the complexities of diet, therapeutics and hospitalisation on gut microbiota. Additionally, while our study has shown that the gut microbiota in CF have increased abundances of pathways and genes, meta-transcriptomics and meta-proteomics would be required to confirm that these genes are more highly expressed in the CF groups compared to non-CF controls.

To conclude, this is the first study to investigate the gut microbiota of adults with CF using shotgun metagenomic sequencing and metabolomics. The results found measurable changes to the gut microbiota phylogeny and functionality in the adult CF samples tested. The results are important and provide insights that can shape larger future studies so that we can continue to appreciate the role of, and the changes to, the gut microbiota in CF.

## Methods

### Participants

Fresh faecal samples were collected from 12 individuals (six patients with CF, collected during pulmonary stability (aged 23–71) and six healthy controls (aged 20–65 years)). See Supplementary Table S1 and S2 for clinical characteristics of the CF group. Controls were healthy adults free from gastrointestinal conditions, with no antibiotic exposure in the 28 days prior to sample collection. Participants provided written informed consent. Four of the six CF patients were pancreatic insufficient. Half of the CF patients were homozygous dF508 and half were heterozygous dF508. Five of the six patients with CF had oral antibiotics in the previous 12 months prior to sample collection (Supplementary Table S2), with the most recent being two weeks prior to sample collection (CF6). All six individuals with CF had IVs in the previous 12 months, ranging from one-five courses of 14 day duration. Five of the six individuals with CF were on long-term oral Azithromycin and all 6 were on nebulized antibiotics (Supplementary Table S2). Ethical approval was received from the Clinical Research Ethics Committee of the Cork Teaching Hospitals, Cork, Ireland and all experiments were performed in accordance with relevant guidelines and regulations. Fresh faecal samples were collected and were immediately frozen at −80 °C between collection and DNA extraction, in line with best practice (as previously determined)^36^ to minimise changes to the microbiota.

### Metagenomic DNA extraction and Nextera XT preparation

Total metagenomic DNA was extracted from the 12 faecal samples as previously described^23, 37^. DNA was quantified using the Qubit in combination with the Invitrogen High Sensitivity DNA assay (BioSciences, Dublin, Ireland). DNA was diluted to 0.2 ng/µl for use as input DNA for Nextera XT based preparation for shotgun metagenomic sequencing on the Illumina MiSeq platform. Briefly, the DNA was firstly tagmented, whereby the DNA was sheared to ~400–600 bp fragments and tagged with Illumina adaptor sequences. An indexing PCR was then completed to individually index each sample to enable pooling of samples on one flow cell and subsequent bioinformatic demultiplexing. PCR amplicons were cleaned and normalised, as per Illumina Nextera XT protocols. To verify the even distribution of samples for pooling, qPCR was performed using the Kapa BioSciences MiSeq qPCR quantification kit (KapaBioSciences, London, UK).

### Illumina MiSeq shotgun sequencing

All metagenomic samples were prepared for sequencing on the Illumina MiSeq platform in the Teagasc sequencing facility. Samples were pooled at a concentration of 4pM and loaded onto the flowcell and sequenced using a 2 × 300 bp V2 Illumina MiSeq sequencing kit. Four samples were sequenced per run, to maximise sequencing depth and coverage.

### Quantitative PCR (qPCR) to quantify total bacteria counts in faecal samples

Total bacterial counts in the faecal samples were determined using qPCR on the Roche Lightcycler 480 (Roche Diagnostics, West Sussex, United Kingdom) as previously described^9^. A standard curve was established using copy numbers of 16S rRNA/μl from 10^9^–10^2^ copies 16S rRNA/μl. The following primers were used: forward primer ACT CCT ACG GGA GGC AGC AG and reverse primer ATT ACC GCG GCT GCT GG. The reaction contained 1 µl DNA, 5 µl Kapa Mastermix (KK4611), 0.2 µl forward primer (10 nM), 0.2 µl reverse primer (10 nM) and 3.6 µl PCR grade water. The following programme was used to quantify total bacterial numbers: denaturation 95 °C × 3 mins followed by amplification of 95 °C × 10S, 60 °C × 20S and 72 °C × 1S for 45 cycles, a melting curve: 95 °C × 5S, 65 °C × 1 min and 97 °C continuous, followed by cooling at 40 °C × 10S.

### Faecal water preparation for GC-MS analysis

Faecal water was prepared from each sample for analysis by GC-MS. Four hundred milligrams of each frozen faecal sample was used. Twice the volume of PBS was added to each faecal aliquot and the sample was homogenized by vortexing. Once completely homogenized, the sample was centrifuged at 16,000 g for 30 mins. The supernatant was removed and centrifuged at 16,000 g × 30 mins to remove faecal solids. This was repeated twice. The supernatant was then filtered through VectaSpin Micro centrifuge filters (0.2 µm) at 10,000 g for 4–6 mins until all the faecal water had passed through the filter. The faecal water was stored at −20 °C until analysis by GC-MS.

### GC-MS analysis

All 12 samples were analysed in triplicate. Prior to analysis, all 36 samples were derivatized with methyl chloroformate and subsequently analysed by GC-MS (MS-omics, Fredericksberg, Denmark). Samples were analysed in a randomised sequence. From each sample, a small aliquot was pooled to a quality control (QC) sample and the QC samples were analysed equally spread throughout the sequence. Relative concentrations of known and unknown compounds were identified using PARAFAC2. Due to the derivatization, some compounds result in more than one peak and the numbers of compounds were therefore lower than the number of resolved peaks. In addition, some peaks originate from impurities in solvents. These peaks were removed from the data resulting in 99 compounds. Compounds included in the standards were quantified. Testing of matrix effects was performed by spiking and dilution of QC samples. It is assumed that the individual sample does not have matrix effects not found in the QC samples.

### Bioinformatic and statistical analysis

#### HUMAnN2 analysis of shotgun data

Raw reads were filtered based on quality, quantity and length of reads with a combination of Picardtools (http://broadinstitute.github.io/picard/) and SAMtools^38^ and human contamination removed with bmtagger (ftp://ftp.ncbi.nlm.nih.gov/pub/agarwala/bmtagger/). Subsequently function was assigned to reads using the HUMAnN2 suite of tools^39^; which assigned function based on the chocophlan databases and genes based on UniRef^40^. The HUMAnN2 gene abundance table was regrouped using a mapping of GO terms that descend from |BP|03|cellular amino acid metabolic process, |BP|03|carbohydrate metabolic processes, |BP|03|lipid metabolic processes and |BP|03|xenobiotics metabolic processes to UniRef families. This was done separately for each of these metabolic processes. MetaPhlAn2 was used to extract taxonomical profiles from the shotgun sequencing results. Graphical representation of the MetaPhlAn2 results were generated using the GraPhlAn software^19, 20, 41^.

### Statistical analysis

Statistical analysis was conducted using the R statistical software package (3.2.2). Non-parametric Kruskall Wallis tests were conducted on the GC-MS metabolite data, pathway abundances and gene family abundances, with p < 0.05 accepted as a significant result (all values have been corrected for multiple testing with the benjamini-hochberg method). Principle coordinate analysis (PCA) and heat maps were generated using the ggplots2, gplots, Rtools, devtools and cluster packages in R. The 3D scatter plots were generated with the following axes: on the x-axis carbohydrate metabolic process = GO:0005975|BP|03|, lipid metabolic process on the y-axis = GO:0006629|BP|03| and as the z-axis xenobiotic metabolic process = GO:0006805|BP|03| or cellular amino acid metabolic process GO:0006520|BP|03|. These were generated using the R statistical packages car and rgl. Procrustes and co-inertia analysis were used to compare metabolomic and metagenomic datasets also in R.

### Data Availability

Data is available through http://www.ebi.ac.uk/ena/data/view/PRJEB21353 with project accession # PRJEB21353.

## Electronic supplementary material

Supplementary information

## References

[1] Foundation, C. F. Cystic Fibrosis Foundation Patient Registry 2012 Annual Data Report (2012).

[2] WHO In *WHO/ECFTN/ICF(M)A/ECFS*.

[3] Davis PB (2006). Cystic fibrosis since 1938. American journal of respiratory and critical care medicine.

[4] Modolell I, Guarner L, Malagelada J-R (2002). Digestive system involvement in cystic fibrosis. Pancreatology.

[5] Rafii F, Sutherland JB, Cerniglia CE (2008). Effects of treatment with antimicrobial agents on the human colonic microflora. Therapeutics and clinical risk management.

[6] Ajslev T, Andersen C, Gamborg M, Sørensen T, Jess T (2011). Childhood overweight after establishment of the gut microbiota: the role of delivery mode, pre-pregnancy weight and early administration of antibiotics. International journal of obesity.

[7] Dethlefsen, L., Huse, S., Sogin, M. L. & Relman, D. A. The pervasive effects of an antibiotic on the human gut microbiota, as revealed by deep 16S rRNA sequencing. *PLoS Biol*. **6**, e280, doi:10.137/journal.pbio.0060280 (2008). 10.1371/journal.pbio.0060280 PMC2586385 19018661

[8] Dethlefsen L, Relman DA (2011). Incomplete recovery and individualized responses of the human distal gut microbiota to repeated antibiotic perturbation. Proceedings of the National Academy of Sciences.

[9] Fouhy F (2012). High-throughput sequencing reveals the incomplete, short-term, recovery of the infant gut microbiota following parenteral antibiotic treatment with ampicillin and gentamycin. Antimicrobial agents and chemotherapy.

[10] Hoen AG (2015). Associations between gut microbial colonization in early life and respiratory outcomes in cystic fibrosis. The Journal of pediatrics.

[11] Scanlan PD (2012). Gut dysbiosis in cystic fibrosis. Journal of Cystic Fibrosis.

[12] Duytschaever G (2011). Cross-sectional and longitudinal comparisons of the predominant fecal microbiota compositions of a group of pediatric patients with cystic fibrosis and their healthy siblings. Applied and environmental microbiology.

[13] Schippa S (2013). Cystic fibrosis transmembrane conductance regulator (CFTR) allelic variants relate to shifts in faecal microbiota of cystic fibrosis patients. PLoS One.

[14] Madan J (2012). Serial analysis of the gut and respiratory microbiome in cystic fibrosis in infancy: interaction between intestinal and respiratory tracts and impact of nutritional exposures. MBio.

[15] Boutin S (2015). Comparison of microbiomes from different niches of upper and lower airways in children and adolescents with cystic fibrosis. PLoS One.

[16] Twomey KB (2013). Microbiota and metabolite profiling reveal specific alterations in bacterial community structure and environment in the cystic fibrosis airway during exacerbation. PloS One.

[17] Quinn RA (2014). Biogeochemical forces shape the composition and physiology of polymicrobial communities in the cystic fibrosis lung. MBio.

[18] Manor O (2016). Metagenomic evidence for taxonomic dysbiosis and functional imbalance in the gastrointestinal tracts of children with cystic fibrosis. Scientific reports.

[19] Asnicar F, Weingart G, Tickle TL, Huttenhower C, Segata N (2015). Compact graphical representation of phylogenetic data and metadata with GraPhlAn. PeerJ.

[20] Segata N (2012). Metagenomic microbial community profiling using unique clade-specific marker genes. Nature methods.

[21] Andersen DH (1938). Cystic fibrosis of the pancreas and its relation to celiac disease: a clinical and pathologic study. American journal of Diseases of Children.

[22] De Vries LE (2011). The gut as reservoir of antibiotic resistance: microbial diversity of tetracycline resistance in mother and infant. PLoS One.

[23] Fouhy F (2014). Identification of aminoglycoside and β-lactam resistance genes from within an Infant gut functional metagenomic library. PLoS One.

[24] Sommer MOA, Dantas G, Church GM (2009). Functional characterization of the antibiotic resistance reservoir in the human microflora. Science.

[25] Duytschaever G, Huys G, Boulanger L, De Boeck K, Vandamme P (2013). Amoxicillin–clavulanic acid resistance in fecal Enterobacteriaceae from patients with cystic fibrosis and healthy siblings. Journal of Cystic Fibrosis.

[26] Arias CA, Murray BE (2009). Antibiotic-resistant bugs in the 21st century—a clinical super-challenge. New England Journal of Medicine.

[27] van Schaik, W*. et al*. Pyrosequencing-based comparative genome analysis of the nosocomial pathogen Enterococcus faecium and identification of a large transferable pathogenicity island. *BMC genomics* **11**, doi:10.1186/1471-2164-11-239 (2010). 10.1186/1471-2164-11-239 PMC2858755 20398277

[28] Willems RJ, Van Schaik W (2009). Transition of Enterococcus faecium from commensal organism to nosocomial pathogen. Future microbiology.

[29] Randazzo A, Kornreich A, Lissoir B (2015). A Clostridium hathewayi isolate in blood culture of a patient with an acute appendicitis. Anaerobe.

[30] Woo PC (2004). Bacteremia due to Clostridium hathewayi in a patient with acute appendicitis. Journal of clinical microbiology.

[31] Ionescu AA (2002). Pulmonary function, body composition, and protein catabolism in adults with cystic fibrosis. American journal of respiratory and critical care medicine.

[32] Debyser G (2015). Faecal proteomics: A tool to investigate dysbiosis and inflammation in patients with cystic fibrosis. Journal of Cystic Fibrosis.

[33] Duytschaever G (2013). Dysbiosis of bifidobacteria and Clostridium cluster XIVa in the cystic fibrosis fecal microbiota. Journal of Cystic Fibrosis.

[34] Consortium HMP (2012). Structure, function and diversity of the healthy human microbiome. Nature.

[35] Lozupone CA, Stombaugh JI, Gordon JI, Jansson JK, Knight R (2012). Diversity, stability and resilience of the human gut microbiota. Nature.

[36] Fouhy F (2015). The Effects of Freezing on Faecal Microbiota as Determined Using MiSeq Sequencing and Culture-Based Investigations. PloS One.

[37] Jones BV, Begley M, Hill C, Gahan CG, Marchesi JR (2008). Functional and comparative metagenomic analysis of bile salt hydrolase activity in the human gut microbiome. Proceedings of the National Academy of Sciences.

[38] Li H (2009). The Sequence Alignment/Map format and SAMtools. Bioinformatics.

[39] Abubucker S (2012). Metabolic reconstruction for metagenomic data and its application to the human microbiome. PLoS Comput Biol.

[40] Suzek BE, Huang H, McGarvey P, Mazumder R, Wu CH (2007). UniRef: comprehensive and non-redundant UniProt reference clusters. Bioinformatics.

[41] Truong DT (2015). MetaPhlAn2 for enhanced metagenomic taxonomic profiling. Nature methods.

