# Supplementary material for: A pilot study demonstrating the altered gut microbiota functionality in stable adults with Cystic Fibrosis
Source: Sci Rep. 2017 Jul 27;7:6685. doi: 10.1038/s41598-017-06880-y (PMC5532234; doi:10.1038/s41598-017-06880-y)
Supplement: Supplementary file 1 — Supplementary information [file 41598_2017_6880_MOESM1_ESM.pdf]

A pilot study demonstrating the altered gut microbiota functionality in stable adults with Cystic Fibrosis

F. Fouhy[1], N.J Ronan[2,3], O. O'Sullivan [1,4], Y. McCarthy[3], A. M. Walsh[1,4], D.M. Murphy [2,3], M. Daly [3], E.T. Flanagan [2,3], C. Fleming[3], M. Mc Carthy[3], C. Shortt [3], J. A. Eustace[2], F. Shanahan [4,5], M.C. Rea[1,4], R. P. Ross [1,4,6], C. Stanton [1,4], B.J. Plant [2,3,4,5*]

[1] Teagasc Food Research Centre, Moorepark, Fermoy, Co. Cork, Ireland
[2] HRB Clinical Research Facility, University College Cork, Cork, Ireland
[3] Cork Cystic Fibrosis Centre, University College Cork, Cork University Hospital, Wilton, Cork, Ireland
[4] APC Microbiome Institute, Cork, Ireland
[5] Department of Medicine, University College Cork, National University of Ireland, Cork,
Ireland
[6]School of Microbiology, University College Cork, Cork, Ireland

Sample ID	Age at sample collection	BMI	% predicted FEV1	Genotype	Pancreatic insufficient	Gender
CF1	30	18	45.79%	dF508/R1066H	Yes	F
CF2	30	21	56.91%	dF508/dF508	Yes	M
CF3	23	20	72.43%	dF508/G551D	Yes	M
CF4	71	24	54.96%	dF508/R117L	No	F
CF5	33	24	80.02%	dF508/dF508	No	M
CF6	29	23	51.93%	dF508/dF508	Yes	F

Supplementary Table S1: Clinical characteristics of the CF group. Gender: F-female; M-male.

CF ID	Oral antibiotics (previous 12 months)	Number of oral antibiotic (previous 12m)	Number of days between last oral antibiotic and sample collection	IV Antibiotics (previous 12m)	Number of IV antibiotics (previous 12m)	Number of days between last IV antibiotic and sample collection	Long term oral antibiotics	Nebulised Antibiotics	NOTES
CF 1	Ciprofloxacin + Co-amoxiclav 22/1/14 x 2 weeks<br>Co-amoxiclav + ciprofloxacin 19/3/14 x 2 weeks	2	281	Meropenem + Tobramycin 22/4/14 x 2 weeks<br>Meropenem + Tobramcyin 17/6/14 x 2 weeks<br>Meropenem + Tobramycin 27/11/14 x 2 weeks	3	28	Azithromycin x long-standing	Colomycin (neb) alternate months<br>Aztreonam alternate months	
CF 2	Ciprofloxacin + Doxycycline 24/4/14 x 2 weeks<br>Ciprofloxacin 14/7/14 x 2 weeks<br>Ciprofloxacin + Doxycycline 22/10/14 x 2 weeks	3	64	Ceftazidime + Tobramycin 24/11/14 x 2 weeks	1	31	Azithromycin x long-standing	Tobramycin (neb) alternate months<br>Aztreonam alternate months	
CF 3	nil	0	N/A	Ceftazidime + Tobramycin 17/7/14 x 2 weeks	1	168	Azithromycin x long-standing	Tobramycin (Tobipodhaler) alternate months<br>Colomycin alternate months	
CF 4	Linezolid + Ciprofloxacin 8/1/14 x 2 weeks<br>Ciprofloxacin + Doxycycline 6/5/14 x 2 weeks	2	240	Ceftazidime 8/1/14 x 2 weeks	1	358	nil	Aztreonam neb alternate months	Immuno-suppression with: Methotrexate, sulphasalaine, entanercept

CF 5	Ciprofloxacin + Doxycyline 24/4/14 x 2 weeks<br>Co-amoxiclav 12/5/14 x 2 weeks<br>Ciprofloxacin 8/9/14 x 2 weeks<br>Ciprofloxacin + Doxycycline Nov 2014 x 2 weeks<br>Ciprofloxacin + Co-trimoxazole 8/12/14 x 2 weeks	5	24	Meropenem + Tobramycin 1/7/14 x 2 weeks	1	184	Azithromycin x long-standing	Tobramycin (Tobipodhaler) alternate months<br>Aztreonam alternate months	
CF 6	Ciprofloxacin + Doxycycline 14/4/14 x 2 weeks<br>Ciprofloxacin + Co-Amoxiclav 17/12/14 x 2 weeks	2	15	Ceftazidime + Tobramycin 3/3/14 x 2 weeks<br>Meropenem + Tobramycin 6/5/14 x 2 weeks<br>Meropenem + Tobramycin 23/6/14 x 2 weeks<br>Ceftazidime + Tobramycin 29/9/14 until 9/10/14<br>Meropenem + Tobramycin 9/10/14 x 2 weeks	5	84	Azithromycin x long-standing	Tobramycin (Tobipodhaler) alternate months<br>Aztreonam alternate months	

Supplementary Table S2: Antibiotic and immunosuppressant medication in the previous 12 months prior to sample collection for the CF cohort

GO functional assignment	Median value for CF group	Median value for control group	p value
**Amino acid metabolism**			
GO:0019441|BP|05|tryptophan catabolic process to kynurenine	333 [250;378]	91.4 [75.9;95.4]	0.004
GO:0018401|BP|05|peptidyl-proline hydroxylation to 4-hydroxy-L-proline	0.12 [0.08;0.16]	0.45 [0.30;0.69]	0.006
GO:0070189|BP|04|kynurenine metabolic process	346 [335;431]	149 [137;162]	0.006
GO:0006562|BP|05|proline catabolic process	222 [173;275]	63.8 [16.4;108]	0.016
GO:0006528|BP|05|asparagine metabolic process	4423 [3574;4963]	2691 [2590;3298]	0.025
GO:0006529|BP|06|asparagine biosynthetic process	4024 [3175;4526]	2468 [2336;3100]	0.025
GO:0006530|BP|06|asparagine catabolic process	22.3 [17.3;24.1]	9.61 [5.38;13.2]	0.025
GO:0010133|BP|06|proline catabolic process to glutamate	199 [155;232]	51.5 [16.0;101]	0.025
GO:0019471|BP|04|4-hydroxyproline metabolic process	12.5 [10.6;15.4]	5.76 [3.98;8.14]	0.025
GO:0033345|BP|07|asparagine catabolic process via L-aspartate	10.0 [7.24;12.9]	2.50 [1.20;3.45]	0.025
GO:0000162|BP|06|tryptophan biosynthetic process	8024 [7442;9241]	6116 [5521;6362]	0.037
GO:0006568|BP|05|tryptophan metabolic process	8623 [7750;9874]	6472 [5721;6695]	0.037
GO:0010121|BP|06|arginine catabolic process to proline via ornithine	10.4 [9.31;18.3]	4.73 [2.23;7.59]	0.037
GO:0019493|BP|05|arginine catabolic process to proline	10.4 [9.31;18.3]	4.73 [2.23;7.59]	0.037
GO:0070981|BP|07|L-asparagine biosynthetic process	2979 [2327;3312]	1990 [1698;2398]	0.037
GO:0070982|BP|06|L-asparagine metabolic process	2979 [2327;3312]	1990 [1698;2398]	0.037
**Lipid metabolism**			
GO:0006633|BP|05|fatty acid biosynthetic process	20027 [17124;22373]	14712 [13021;15649]	0.016
GO:0046488|BP|06|phosphatidylinositol metabolic process	971 [791;1018]	378 [177;570]	0.016
GO:0046834|BP|05|lipid phosphorylation	967 [789;1010]	386 [173;602]	0.016
GO:0046854|BP|06|phosphatidylinositol phosphorylation	940 [783;995]	357 [158;542]	0.016
GO:0033542|BP|07|fatty acid beta-oxidation, unsaturated, even number	0.00 [0.00;0.00]	0.10 [0.05;0.13]	0.021
GO:0097089|BP|05|methyl-branched fatty acid metabolic process	0.07 [0.01;0.30]	0.00 [0.00;0.00]	0.022
GO:0006631|BP|04|fatty acid metabolic	24222	18177 [17387;19902]	0.025

process	[20460;27343]		
GO:0006655|BP|06|phosphatidylglycerol biosynthetic process	2036 [1767;2293]	1358 [1181;1547]	0.025
GO:0006665|BP|04|sphingolipid metabolic process	1035 [775;1483]	472 [438;706]	0.025
GO:0008653|BP|04|lipopolysaccharide metabolic process	8867 [7912;10182]	6459 [5717;6717]	0.025
GO:0009103|BP|05|lipopolysaccharide biosynthetic process	8867 [7912;10176]	6458 [5717;6717]	0.025
GO:0030497|BP|06|fatty acid elongation	1177 [1026;1230]	795 [692;897]	0.025
GO:0032048|BP|07|cardiolipin metabolic process	1740 [1423;2016]	887 [644;1297]	0.025
GO:0032049|BP|07|cardiolipin biosynthetic process	1740 [1422;2016]	886 [643;1297]	0.025
GO:0046471|BP|06|phosphatidylglycerol metabolic process	2037 [1768;2294]	1360 [1183;1547]	0.025
GO:0046360|BP|07|2-oxobutyrate biosynthetic process	0.00 [0.00;0.09]	0.26 [0.09;0.73]	0.034
GO:0046361|BP|06|2-oxobutyrate metabolic process	0.00 [0.00;0.09]	0.26 [0.09;0.73]	0.034
GO:0006642|BP|07|triglyceride mobilization	1.24 [0.72;2.01]	3.41 [3.05;5.00]	0.037
GO:0006700|BP|04|C21-steroid hormone biosynthetic process	0.30 [0.08;0.68]	0.00 [0.00;0.05]	0.046
GO:0006701|BP|05|progesterone biosynthetic process	0.30 [0.08;0.68]	0.00 [0.00;0.05]	0.046
GO:0016254|BP|06|preassembly of GPI anchor in ER membrane	0.00 [0.00;0.08]	0.11 [0.10;0.20]	0.05
**Carbohydrate metabolism**			
GO:0005997|BP|06|xylulose metabolic process	712 [532;870]	232 [185;294]	0.004
GO:0006014|BP|06|D-ribose metabolic process	1902 [1684;2273]	558 [495;623]	0.004
GO:0019299|BP|06|rhamnose metabolic process	3385 [3229;3782]	1656 [1536;2471]	0.004
GO:0019400|BP|03|alditol metabolic process	5777 [5209;6120]	3252 [3030;3374]	0.004
GO:0019852|BP|05|L-ascorbic acid metabolic process	636 [598;816]	310 [226;371]	0.004
GO:0019854|BP|05|L-ascorbic acid catabolic process	609 [555;787]	259 [188;324]	0.004
GO:0036065|BP|05|fucosylation	301 [225;384]	95.6 [46.0;149]	0.004
GO:0042836|BP|06|D-glucarate metabolic process	264 [154;434]	59.2 [50.4;74.6]	0.004
GO:0042838|BP|07|D-glucarate catabolic process	264 [154;434]	59.2 [50.4;74.6]	0.004
GO:0005975|BP|03|carbohydrate metabolic process	215916 [200986;244914]	125668 [116396;144120]	0.006
GO:0010412|BP|05|mannan metabolic process	683 [657;846]	108 [69.3;210]	0.006

GO:0019303|BP|07|D-ribose catabolic process	796 [605;912]	158 [136;196]	0.006
GO:0019321|BP|05|pentose metabolic process	7844 [6849;9257]	3209 [2917;4367]	0.006
GO:0019520|BP|04|aldonic acid metabolic process	790 [574;1029]	355 [228;391]	0.006
GO:0019521|BP|05|D-gluconate metabolic process	739 [547;892]	315 [181;335]	0.006
GO:0019577|BP|04|aldaric acid metabolic process	585 [350;820]	167 [128;213]	0.006
GO:0019579|BP|05|aldaric acid catabolic process	585 [350;820]	167 [128;213]	0.006
GO:0046355|BP|05|mannan catabolic process	683 [657;846]	107 [68.8;210]	0.006
GO:0046365|BP|05|monosaccharide catabolic process	23393 [22201;30463]	17458 [16743;19317]	0.006
GO:0006004|BP|06|fucose metabolic process	4780 [4213;5059]	1981 [1859;2328]	0.01
GO:0006063|BP|05|uronic acid metabolic process	3772 [3014;4308]	2173 [1678;2486]	0.01
GO:0006080|BP|05|substituted mannan metabolic process	455 [302;667]	103 [72.9;137]	0.01
GO:0019323|BP|06|pentose catabolic process	2246 [2169;2605]	1201 [939;1586]	0.01
GO:0019698|BP|07|D-galacturonate catabolic process	552 [426;731]	247 [152;273]	0.01
GO:0042120|BP|04|alginic acid metabolic process	1227 [982;1465]	401 [332;530]	0.01
GO:0042121|BP|05|alginic acid biosynthetic process	1227 [982;1465]	401 [332;530]	0.01
GO:0042353|BP|07|fucose biosynthetic process	160 [134;164]	110 [102;122]	0.01
GO:0044247|BP|05|cellular polysaccharide catabolic process	3919 [3706;4001]	2231 [2037;2654]	0.01
GO:0044275|BP|04|cellular carbohydrate catabolic process	8042 [7524;8323]	5019 [4694;5617]	0.01
GO:0046396|BP|07|D-galacturonate metabolic process	552 [426;731]	247 [152;273]	0.01
GO:0046835|BP|04|carbohydrate phosphorylation	11591 [10484;13607]	6794 [6614;8088]	0.01
GO:0005996|BP|04|monosaccharide metabolic process	53533 [48054;63494]	39243 [37801;41579]	0.016
GO:0006098|BP|05|pentose-phosphate shunt	10598 [9186;12580]	8290 [7823;8789]	0.016
GO:0044262|BP|03|cellular carbohydrate metabolic process	54658 [47114;61738]	37217 [36561;37919]	0.016
GO:0044723|BP|03|single-organism carbohydrate metabolic process	119183 [105710;137532]	87237 [84133;91911]	0.016
GO:0052695|BP|07|cellular glucuronidation	0.17 [0.04;0.47]	2.74 [1.53;5.10]	0.016
GO:0000271|BP|05|polysaccharide	24695	18456 [17666;18686]	0.025

biosynthetic process	[22721;27868]		
GO:0005983|BP|06|starch catabolic process	1441 [1156;1985]	531 [393;791]	0.025
GO:0005991|BP|04|trehalose metabolic process	779 [679;1062]	181 [103;270]	0.025
GO:0006039|BP|05|cell wall chitin catabolic process	0.13 [0.01;0.21]	0.52 [0.43;0.72]	0.025
GO:0008653|BP|04|lipopolysaccharide metabolic process	8867 [7912;10182]	6459 [5717;6717]	0.025
GO:0009103|BP|05|lipopolysaccharide biosynthetic process	8867 [7912;10176]	6458 [5717;6717]	0.025
GO:0009243|BP|05|O antigen biosynthetic process	3064 [2966;3659]	2183 [1823;2571]	0.025
GO:0010325|BP|06|raffinose family oligosaccharide biosynthetic process	1.99 [1.83;3.56]	0.16 [0.04;0.50]	0.025
GO:0016052|BP|04|carbohydrate catabolic process	65922 [59571;79747]	49179 [46830;53605]	0.025
GO:0019317|BP|07|fucose catabolic process	2072 [1526;2471]	1266 [980;1498]	0.025
GO:0033531|BP|05|stachyose metabolic process	1.99 [1.83;3.56]	0.16 [0.04;0.50]	0.025
GO:0033532|BP|06|stachyose biosynthetic process	1.99 [1.83;3.56]	0.16 [0.04;0.50]	0.025
GO:0034218|BP|03|ascospore wall chitin metabolic process	0.13 [0.01;0.21]	0.52 [0.43;0.72]	0.025
GO:0034232|BP|04|ascospore wall chitin catabolic process	0.13 [0.01;0.21]	0.52 [0.43;0.72]	0.025
GO:0044264|BP|04|cellular polysaccharide metabolic process	28233 [24833;32382]	21041 [20388;22244]	0.025
GO:0046402|BP|05|O antigen metabolic process	3064 [2966;3659]	2183 [1823;2571]	0.025
GO:0005976|BP|04|polysaccharide metabolic process	40290 [35993;42926]	26028 [24823;27706]	0.037
GO:0005992|BP|05|trehalose biosynthetic process	241 [189;318]	42.1 [11.1;122]	0.037
GO:0005993|BP|05|trehalose catabolic process	128 [78.7;216]	42.7 [31.7;53.8]	0.037
GO:0006013|BP|06|mannose metabolic process	936 [822;1048]	235 [204;499]	0.037
GO:0009052|BP|06|pentose-phosphate shunt, non-oxidative branch	484 [419;727]	196 [117;266]	0.037
GO:0019301|BP|07|rhamnose catabolic process	2488 [2271;2604]	1172 [1106;1672]	0.037
GO:0019318|BP|05|hexose metabolic process	42911 [36535;47970]	32856 [31920;35423]	0.037
GO:0019320|BP|06|hexose catabolic process	17978 [15369;20906]	13990 [12888;15708]	0.037
GO:0033692|BP|04|cellular polysaccharide biosynthetic process	19495 [18011;24220]	17052 [15244;17525]	0.037
GO:0034637|BP|03|cellular carbohydrate	20353	17275 [15886;17766]	0.037

biosynthetic process	[18572;24783]		
GO:0042839|BP|07|D-glucuronate metabolic process	56.3 [50.8;77.0]	32.1 [25.7;36.3]	0.037
GO:0042840|BP|07|D-glucuronate catabolic process	56.3 [50.8;77.0]	32.1 [25.7;36.3]	0.037
GO:0044724|BP|04|single-organism carbohydrate catabolic process	55615 [49087;65395]	41727 [40412;46686]	0.037
GO:0045226|BP|05|extracellular polysaccharide biosynthetic process	6204 [5325;6587]	3832 [3384;4179]	0.037
GO:0045488|BP|06|pectin metabolic process	1642 [1583;1690]	859 [690;1438]	0.037
GO:0045490|BP|06|pectin catabolic process	1603 [1489;1665]	741 [620;1348]	0.037
GO:0046351|BP|04|disaccharide biosynthetic process	266 [210;332]	48.6 [14.2;129]	0.037
GO:0046379|BP|05|extracellular polysaccharide metabolic process	6211 [5330;6594]	3842 [3398;4188]	0.037
GO:0005989|BP|05|lactose biosynthetic process	6.34 [1.90;25.7]	0.02 [0.00;1.13]	0.042
**Xenobiotic degradation**			
GO:0006805|BP|03|xenobiotic metabolic process	1592 [1162;1868]	834 [517;940]	0.01
GO:0042178|BP|04|xenobiotic catabolic process	1117 [786;1588]	448 [261;638]	0.01
GO:0010124|BP|05|phenylacetate catabolic process	790 [617;1408]	263 [157;328]	0.016
GO:0019380|BP|05|3-phenylpropionate catabolic process	30.8 [13.0;48.8]	4.99 [3.47;8.36]	0.025

Supplementary Table S3: Statistical analysis on gene family abundances involved in amino acid, lipid, carbohydrate and xenobiotic metabolic processes using Kruskal Wallis statistical test where significance was accepted as a corrected p value of <0.05.

Metabolite	CF group median	Control group median	p value
X147	-0.05 [-0.06;-0.02]	0.49 [0.22;1.14]	0.004
Pentanedioic acid, 3-hydroxy-3-methyl-	1.23 [0.84;1.69]	0.60 [0.57;0.62]	0.006
Ornithine	0.13 [0.11;0.32]	0.04 [0.03;0.04]	0.006
Tryptophan	0.05 [0.04;0.06]	0.03 [0.02;0.03]	0.006
Dimethyl phthalate	1.23 [0.66;2.62]	0.01 [0.01;0.01]	0.006
X96	0.98 [0.12;2.40]	0.03 [0.02;0.05]	0.01
X151	1.27 [1.18;1.38]	0.87 [0.82;1.03]	0.01
Benzenepropanoic acid, 3,4-dimethoxy-	1.43 [0.76;1.70]	0.40 [0.30;0.48]	0.016
2-Piperidinone	1.38 [0.84;2.46]	0.11 [0.09;0.49]	0.025
1,4-Benzenedicarboxylic acid, 2-amino-,	0.66 [0.59;0.76]	0.52 [0.49;0.58]	0.025
9-Octadecenoic acid	1.56 [0.86;1.65]	0.41 [0.32;0.45]	0.025
Glutamine	0.54 [0.28;1.80]	0.17 [0.12;0.23]	0.037
Myristic acid	1.37 [1.20;1.59]	1.03 [0.87;1.07]	0.037
Hexadecanoic acid	1.53 [1.28;1.77]	0.78 [0.76;0.81]	0.037
Lysine	0.50 [0.38;0.68]	0.21 [0.18;0.36]	0.037
Citric acid	0.07 [0.06;0.11]	0.05 [0.04;0.06]	0.037

Supplementary Table S4: Significant differences in the metabolites present in the CF and control groups based on the GC-MS data.

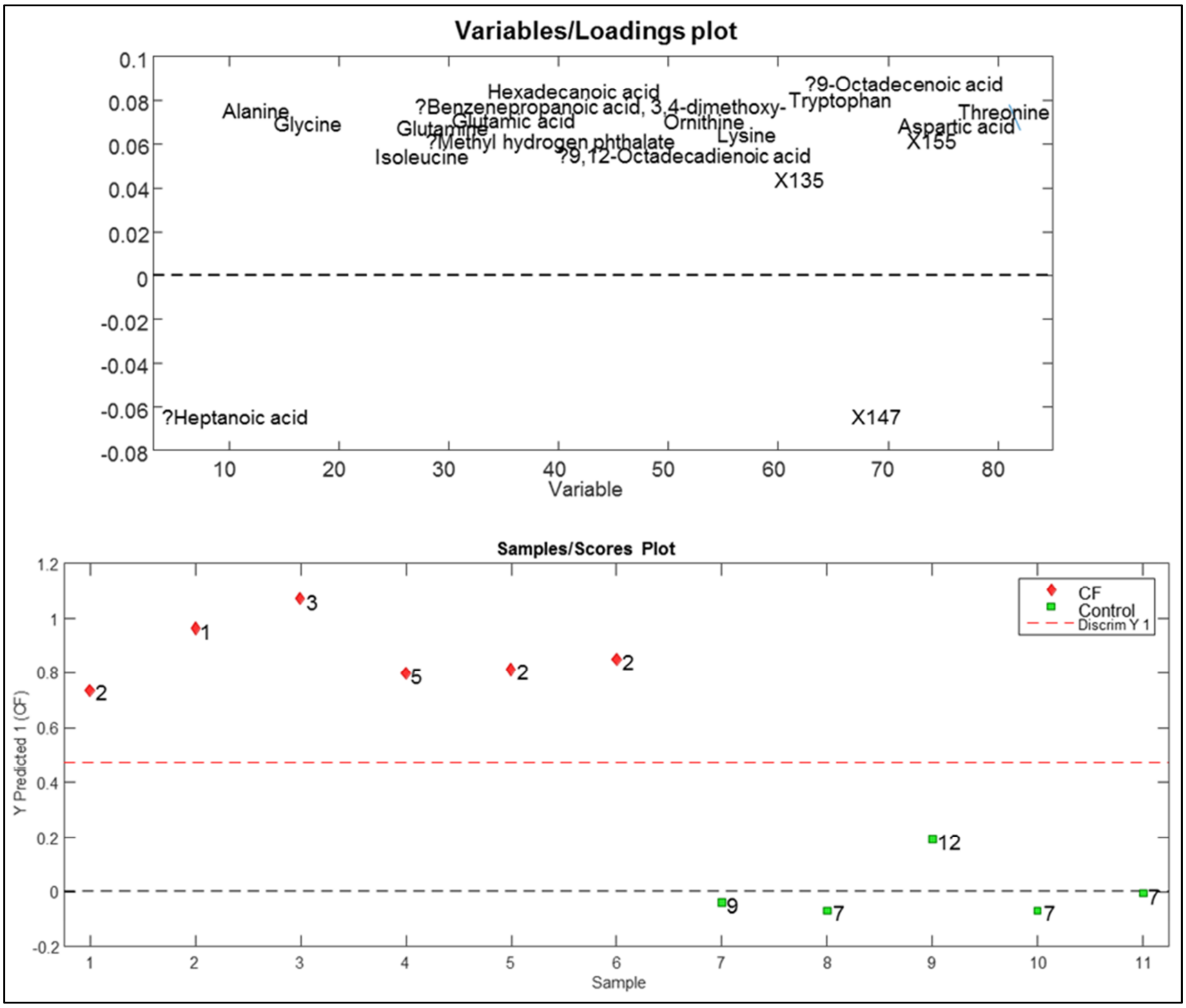

Supplementary Figure S1: Upper plot: regression vector from the PLS-DA model. Lower plot: results from prediction of test-set samples (samples not included in the creation of the model). Upper plot shows metabolites higher in the CF samples when above the 0, and those lower in the CF samples compared to the controls below 0. The lower plot shows correct prediction of whether samples are CF or controls based on PLS-DA model.

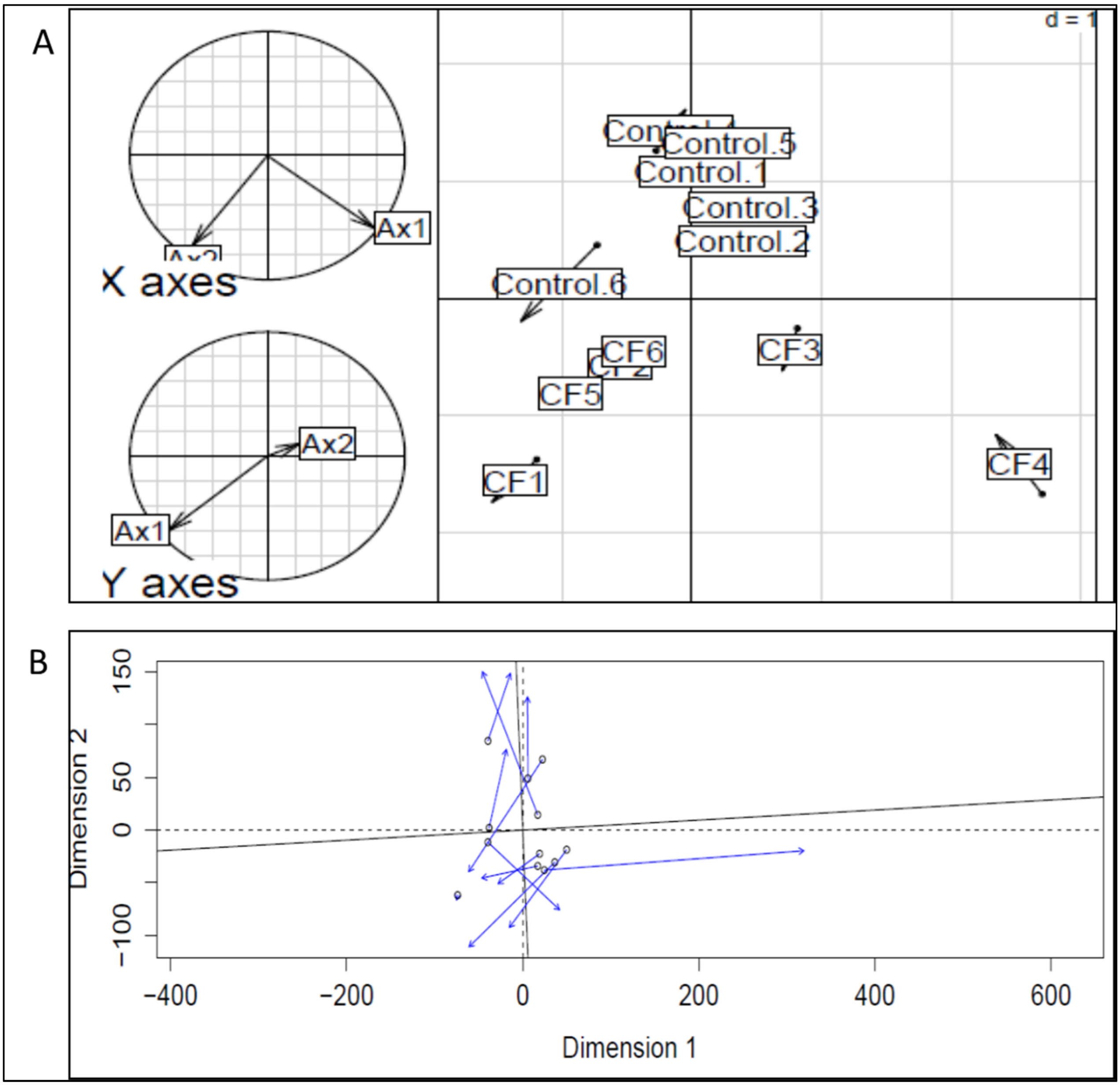

Supplementary Figure S2: Co-inertia (A) and Procrustes analysis (B) both depicted a strong positive correlation (r^2=0.81) between the metabolites and the pathway abundances
